# Protein expression profiling identifies a prognostic model for ovarian cancer

**DOI:** 10.1186/s12905-022-01876-x

**Published:** 2022-07-15

**Authors:** Luyang Xiong, Jiahong Tan, Yuchen Feng, Daoqi Wang, Xudong Liu, Yun Feng, Shusheng Li

**Affiliations:** 1 Department of Critical Care Medicine, Tongji Hospital, Tongji Medical College, Huazhong University of Science and Technology, Wuhan, China; 2 Department of Obstetrics and Gynecology, National Key Clinical Specialty of Gynecology, The First People’s Hospital of Yunnan Province, The Affiliated Hospital of Kunming University of Science and Technology, Kunming, China; 3 Division of Pulmonary and Critical Care Medicine, Department of Internal Medicine, Tongji Hospital, Tongji Medical College, Huazhong University of Science and Technology, Wuhan, China; 4 Department of Urology, The Second Affiliated Hospital of Kunming Medical University, Kunming, China; 5 Department of Pancreatic Surgery, Wuhan Union Hospital, Tongji Medical College, Huazhong University of Science and Technology, Wuhan, China

**Keywords:** Ovarian cancer, Prognosis, Risk model, Survival

## Abstract

**Background:**

Owing to the high morbidity and mortality, ovarian cancer has seriously endangered female health. Development of reliable models can facilitate prognosis monitoring and help relieve the distress.

**Methods:**

Using the data archived in the TCPA and TCGA databases, proteins having significant survival effects on ovarian cancer patients were screened by univariate Cox regression analysis. Patients with complete information concerning protein expression, survival, and clinical variables were included. A risk model was then constructed by performing multiple Cox regression analysis. After validation, the predictive power of the risk model was assessed. The prognostic effect and the biological function of the model were evaluated using co-expression analysis and enrichment analysis.

**Results:**

394 patients were included in model construction and validation. Using univariate Cox regression analysis, we identified a total of 20 proteins associated with overall survival of ovarian cancer patients (*p* < 0.01). Based on multiple Cox regression analysis, six proteins (GSK3α/β, HSP70, MEK1, MTOR, BAD, and NDRG1) were used for model construction. Patients in the high-risk group had unfavorable overall survival (*p* < 0.001) and poor disease-specific survival (*p* = 0.001). All these six proteins also had survival prognostic effects. Multiple Cox regression analysis demonstrated the risk model as an independent prognostic factor (*p* < 0.001). In receiver operating characteristic curve analysis, the risk model displayed higher predictive power than age, tumor grade, and tumor stage, with an area under the curve value of 0.789. Analysis of co-expressed proteins and differentially expressed genes based on the risk model further revealed its prognostic implication.

**Conclusions:**

The risk model composed of GSK3α/β, HSP70, MEK1, MTOR, BAD, and NDRG1 could predict survival prognosis of ovarian cancer patients efficiently and help disease management.

**Supplementary Information:**

The online version contains supplementary material available at 10.1186/s12905-022-01876-x.

## Background

Ovarian cancer, with a mortality-to-incidence ratio exceeding 0.6, has the highest mortality among all malignancies of the female genital system and accounts for more deaths than any other gynecological cancers [1]. Among all cancers threatening women’s health, ovarian cancer ranks fifth in cancer deaths [2]. Annually, there are 239,000 new ovarian cancer cases worldwide, making up 3.6% of all cancer cases, and globally, the incidence has been increasing in most countries [1, 2]. Owing to the lack of specific warning manifestations in the early stage, the majority of ovarian cancer patients are diagnosed at advanced stages, for which the five-year survival rate is estimated below 30% [1]. The asymptomatic progression has seriously worsened the prognosis [1]. Under this circumstance, it is imperative to delineate prognostic biomarkers to relieve the distress in the aggressive disease.

Identification of reliable predictive methods could facilitate disease management and guide prognosis monitoring [3]. Clinical features such as age, stage, grade, and some serum makers are generally and widely used to predict the prognosis of ovarian cancer patients [4–6]. However, they cannot fully reflect tumor biological characteristics, and individual differences lead to inadequate specificity and incompetence [4, 5]. In the past few decades, the rapid and prosperous development of sequencing technology has provided opportunities to systematically explore tumors, and a myriad of genes including RAD51 and PAWR were explored as predictive biomarkers in ovarian cancer [5, 7, 8]. Due to the intratumor heterogeneity of this deadly disease and the complex molecular mechanisms affecting its prognosis, single factor prediction models usually have low accuracy and efficacy [4, 5]. Recently, some gene signatures have been studied to promote prognosis management in various cancers [4]. For example, a panel of 21 genes has been explored in breast cancer to predict disease recurrence [9]. A model consisting of 18 genes was reported to be capable of monitoring recurrence in colon cancer [10]. In ovarian cancer, a transcriptome-based signature was found to affect chemotherapy response [11]. Besides, researchers have also dissected a lncRNA panel to predict the survival and therapeutic responsiveness of ovarian cancer [12]. Definition of new biomarkers using next generation sequencing technology can facilitate molecular targets therapy [13]. Using the multi-omics data to develop multiple-gene-based models could better describe the molecular biological features of ovarian cancer and help predict its prognosis more efficiently [4, 5].

In the present study, proteins having significant survival effects on ovarian cancer patients were screened by univariate Cox regression analysis, and a risk model was then developed using multiple Cox regression method. After validation, the predictive power of the risk model was analyzed and evaluated. The prognostic effect of the proteins comprising the risk model was also explored. Finally, functional analysis based on the risk model was performed and the prognostic value of the proteins comprising the risk model was also delineated.

## Methods

### Data acquisition

Protein expression data of ovarian cancer patients were obtained from The Cancer Proteome Atlas (TCPA) database (https://tcpaportal.org/tcpa/) [14]. Survival data, clinical data, and RNA sequencing data were retrieved from The Cancer Genome Atlas (TCGA) program data portal (https://portal.gdc.cancer.gov/). 411 records were downloaded from the TCPA data portal, which represents 411 unique ovarian cancer samples. Correspondingly, 587 records were retrieved from the TCGA Ovarian Cancer collection. Then the TCPA data were merged with the TCGA data by the unique sample ID. Information concerning protein expression, survival, and clinical features was integrated using the unique sample ID as the identifier, and samples with incomplete information were excluded from downstream analysis. After the merge, we obtained 394 unique records in total.

### Construction of the risk model and survival analysis

Univariate Cox regression was performed to identify proteins associated with patients’ overall survival (OS) with a threshold p value of 0.01. These proteins were subsequently used to develop the risk model, which was then trained and optimized using the multiple Cox regression model of the survival package in R programming language (version: 4.0.5) [15]. A total of 394 ovarian cancer patients were equally and randomly divided into the training set and the validation set, which was used for the development and verification of our risk model correspondingly. The risk score was calculated using the formula below:

$${\text{Risk}}\;{\text{score }} = \mathop \sum \limits_{1}^{n} coef\left( i \right)*expr\left( i \right)$$

where *i* referred to the protein in the risk model, *coef*(*i*) was the coefficient of protein in the model, and *expr*(*i*) represented the expression of protein *i*. Based on the median risk score, patients in the training set were subdivided into a high-risk group and a low-risk group. The predictive value of risk score on OS was assessed using Kaplan–Meier analysis.

### Validation of the risk model and survival analysis

The validation set and the entire patient group were used to confirm the predictive value of the risk model. Patients were classified by median risk score, and the predictive power of risk score on OS and disease-specific survival (DSS) was evaluated using Kaplan–Meier plot. Besides, univariate and multiple Cox regression analyses were performed to evaluate the predictive effect of risk score and other clinical variables (age, tumor stage, and tumor grade) on OS. The receiver operating characteristic curve (ROC) was also plotted to evaluate and compare the predictive accuracy of the risk model by using the survivalROC package [16]. Furthermore, patients were then stratified by age, tumor grade, and tumor stage, and the predictive power of the risk model on survival was also analyzed in the subgroups.

### Analysis of co-expressed proteins, differentially expressed genes, and enriched pathways

Proteins, whose expression was highly correlated with the expression of proteins comprising the risk model, were identified by Pearson’s correlation test. Genes encoding these co-expressed proteins were used for pathway enrichment analyses in the Gene Ontology (GO) or the Kyoto Encyclopedia of Genes and Genomes (KEGG) databases [17–21]. Pathway enrichment analyses were conducted using the R package clusterProfiler [22]. All ovarian cancer patients were further divided into a high-risk group and a low-risk group based on risk score, and differentially expressed genes between these two groups were identified using the limma package [23].

### Statistical analysis

Statistical analysis of bioinformatics was performed as aforementioned. In Kaplan–Meier survival analysis, log-rank test was conducted. Significance was defined at the level of *p* < 0.05 unless otherwise indicated.

## Results

### Construction and validation of the risk model

**Fig. 1:**
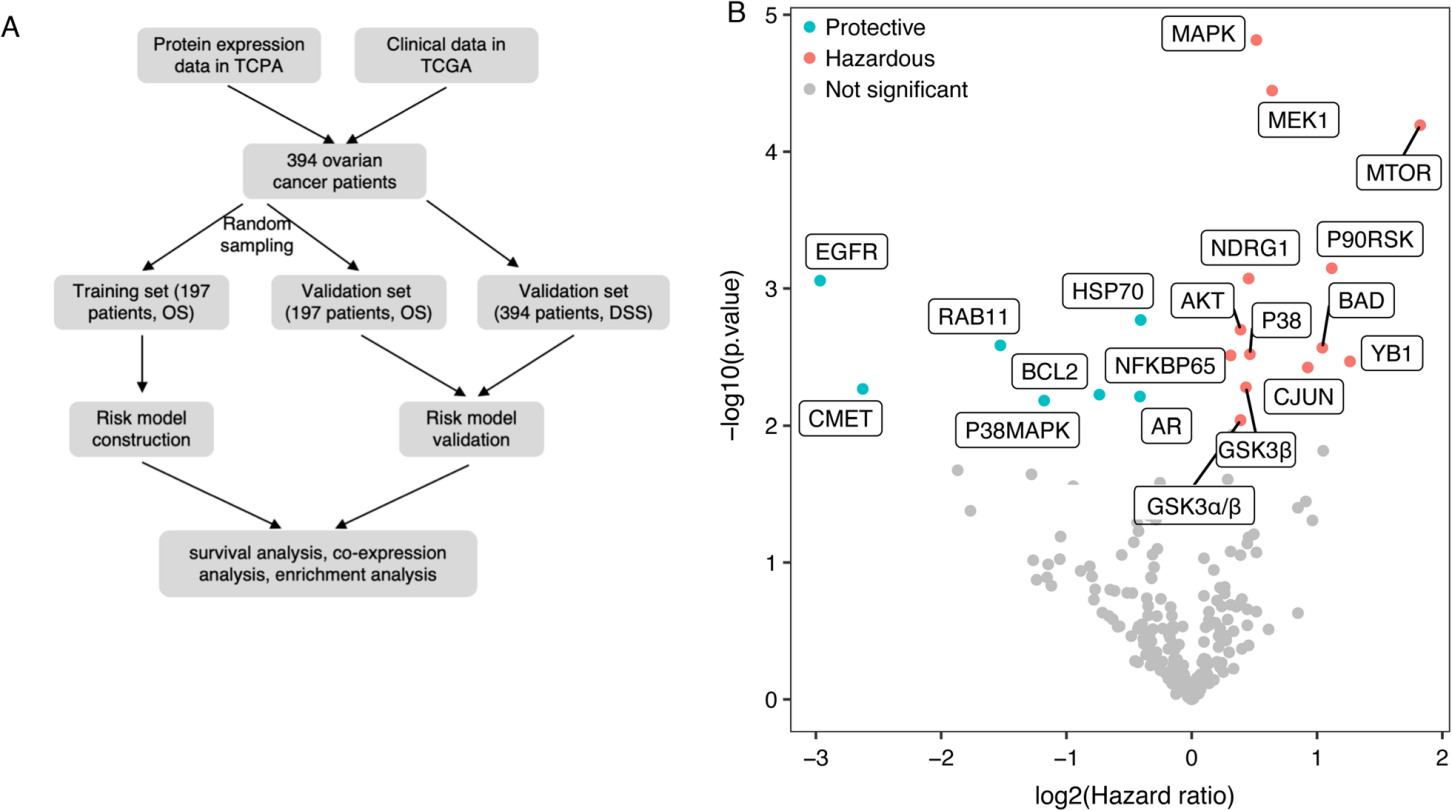
Study design and univariate Cox regression analysis for risk model construction. **A** Flow chart of study design. **B** Volcano plot of proteins identified in univariate Cox regression analysis (*p* < 0.01). DSS, disease-specific survival; OS, overall survival; TCGA, The Cancer Genome Atlas; TCPA, The Cancer Proteome Atlas

Taking advantage of the protein expression information of various cancers archived in the TCPA portal, we obtained the protein expression profile of ovarian cancer patients. The survival data, clinical characteristics, and RNA sequencing data of ovarian cancer patients were obtained from the TCGA database. All retrieved data were integrated for quality check. Samples with incomplete information were filtered, and 394 ovarian cancer patients were included for downstream analysis. As shown in the flow chart (Fig. 1A), these 394 patients were subdivided equally and randomly into a training set and a validation set for model construction, optimization, and verification.

Using univariate Cox regression analysis, a total of 20 proteins was found to significantly affect OS of ovarian cancer patients (*p* < 0.01) (Fig. 1B). Among the 20 proteins, seven of them could be protective factors for OS including HSP70 and BCL2, while the other 13 proteins could deteriorate survival outcome of ovarian cancer patients, including MAPK, MEK1, MTOR, NDRG1, BAD, and GSK3α/β. These 20 proteins were then analyzed using multiple Cox regression method to develop a risk model for OS of ovarian cancer patients. After training and optimization in the training set, six proteins, namely, GSK3α/β, HSP70, MEK1, MTOR, BAD, and NDRG1, were maintained (Table 1).

**Table 1:** Multiple Cox regression result

Protein	Coefficient	HR	*p*
GSK3α/β	− 0.43	0.65 (0.44, 0.97)	0.036
HSP70	− 0.306	0.74 (0.56, 0.98)	0.034
MEK1	0.768	2.16 (1.34, 3.46)	0.001
MTOR	1.18	3.26 (0.99, 10.7)	0.053
BAD	− 0.878	0.42 (0.16, 1.11)	0.079
NDRG1	0.225	1.25 (0.94, 1.67)	0.127

*HR* hazard ratio

**Fig. 2:**
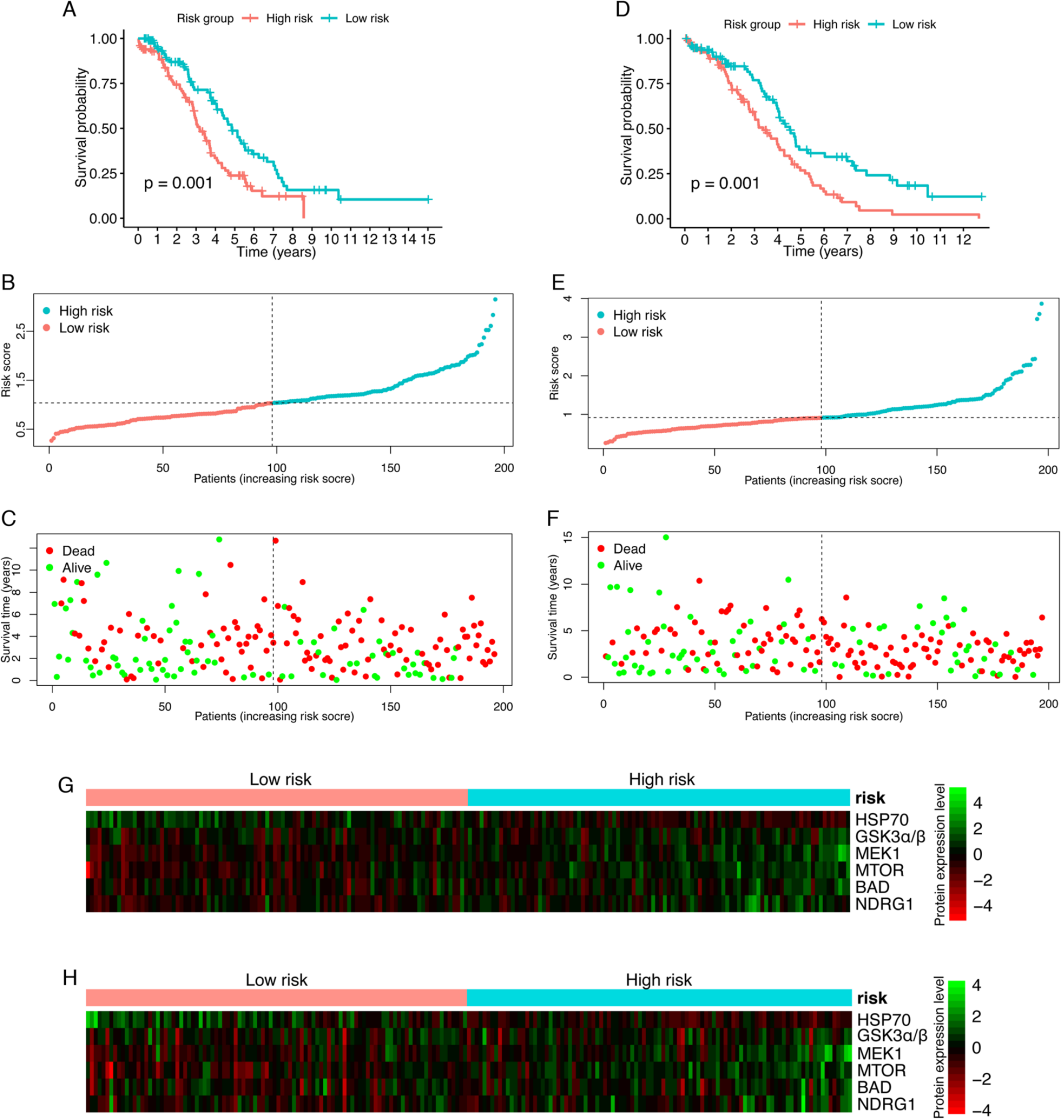
Evaluation of the performance of the risk model. Ovarian cancer patients were divided into a high-risk group and a low-risk group based on the median risk score. **A** Kaplan–Meier plot for OS of ovarian cancer patients in the training set (log-rank test). **B** Distribution of risk score of ovarian cancer patients in the training set. **C** Survival status scatter plot of ovarian cancer patients in the training set. **D** Survival analysis for OS of ovarian cancer patients in the validation set (log-rank test). **E** The risk score distribution of ovarian cancer patients in the validation set. **F** Scatter diagram of survival status of ovarian cancer patients in the validation set. **G** Heatmap of expression profiles of the six genes comprising the risk model in ovarian cancer patients of the training set. **H** The expression patterns of the six proteins comprising the risk model in the validation set

After the construction of the risk model, a risk score was calculated and assigned to each patient. The training set was further divided into a high-risk group and a low-risk group based on the median cutoff risk score. By survival analysis, the high-risk group had shorter OS times (*p* = 0.001) (Fig. 2A). The risk distribution and survival status of the training set were also described in detail (Fig. 2B, C). The risk model was validated in the validation set and the entire patient group afterward. In the validation set, patients with risk scores above the median had a poorer survival prognosis (*p* = 0.001) (Fig. 2D). Consistent results were observed in the entire patient group, wherein an increased risk score predicted unfavorable survival outcomes (Additional file 1: Fig. S1A). The risk distribution and survival status of the validation set and all included patients were further depicted in scatter plots (Fig. 2E, F; Additional file 1: Fig. S1B, C). To get a general insight into the expression profile of the six components of the risk model, their expression was denoted using heatmap in the training set, the validation set, and all patients separately (Fig. 2G, H; Additional file 1: Fig. S1D).

### Prognostic effect of the proteins comprising the risk model

**Fig. 3:**
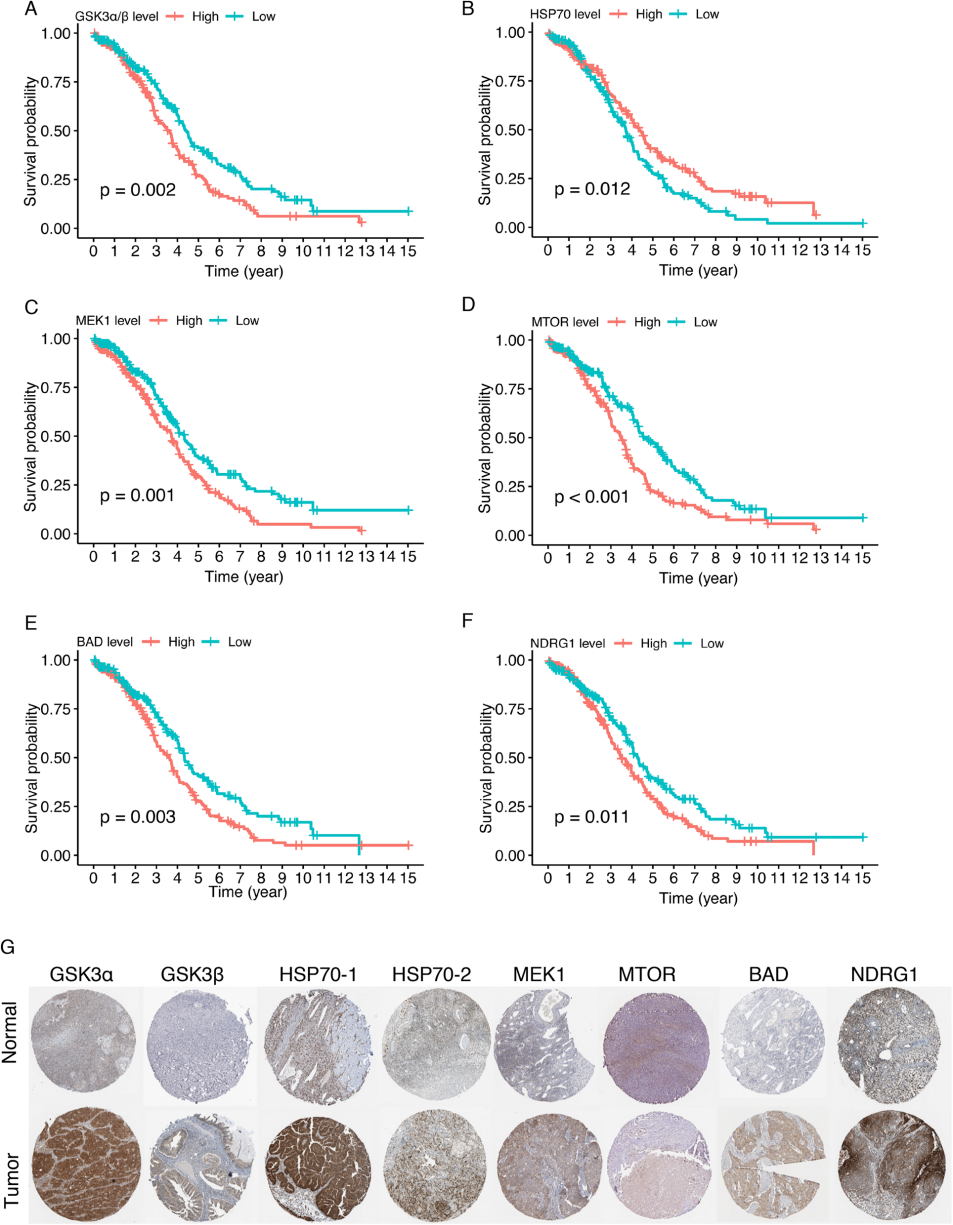
Survival analysis of the proteins comprising the risk model. Kaplan–Meier survival curves of OS comparing the high and low levels of the six proteins comprising the risk model (log-rank test). **A** GSK3α/β. **B** HSP70. **C** MEK1. **D** MTOR. **E** BAD. **F** NDRG1. **G** Representative immunohistochemical staining images of the six proteins comprising the risk model in normal ovary tissue and ovarian cancer tissue in the Human Protein Atlas

We then analyzed the prognostic effect of the six proteins on OS of ovarian cancer patients. High expression of GSK3α/β was pernicious to OS (*p* = 0.002) (Fig. 3A), while high expression of HSP70 was beneficial (*p* = 0.012) (Fig. 3B). By Kaplan–Meier survival analysis, elevated expression of MEK1 (*p* = 0.001), MTOR (*p* < 0.001), BAD (*p* = 0.003), and NDRG1 (*p* = 0.011) indicated unfavorable survival outcome (Fig. 3C–F). We observed consistent results when analyzing the DSS of ovarian cancer patients. Similarly, patients in the high-risk group exhibited shorter DSS (*p* = 0.001) (Additional file 2: Fig S2A). Low expression of GSK3α/β, MEK1, MTOR, BAD, and NDRG1 predicted longer DSS times, while low HSP70 expression suggested shorter DSS times (Additional file 2: Fig. S2B–G). These six proteins were also detected immunohistochemically in the Human Protein Atlas database, and representative images of normal ovary tissues and ovarian cancer tissues were shown (Fig. 3G).

### Prognostic value of the risk model on survival

**Fig. 4:**
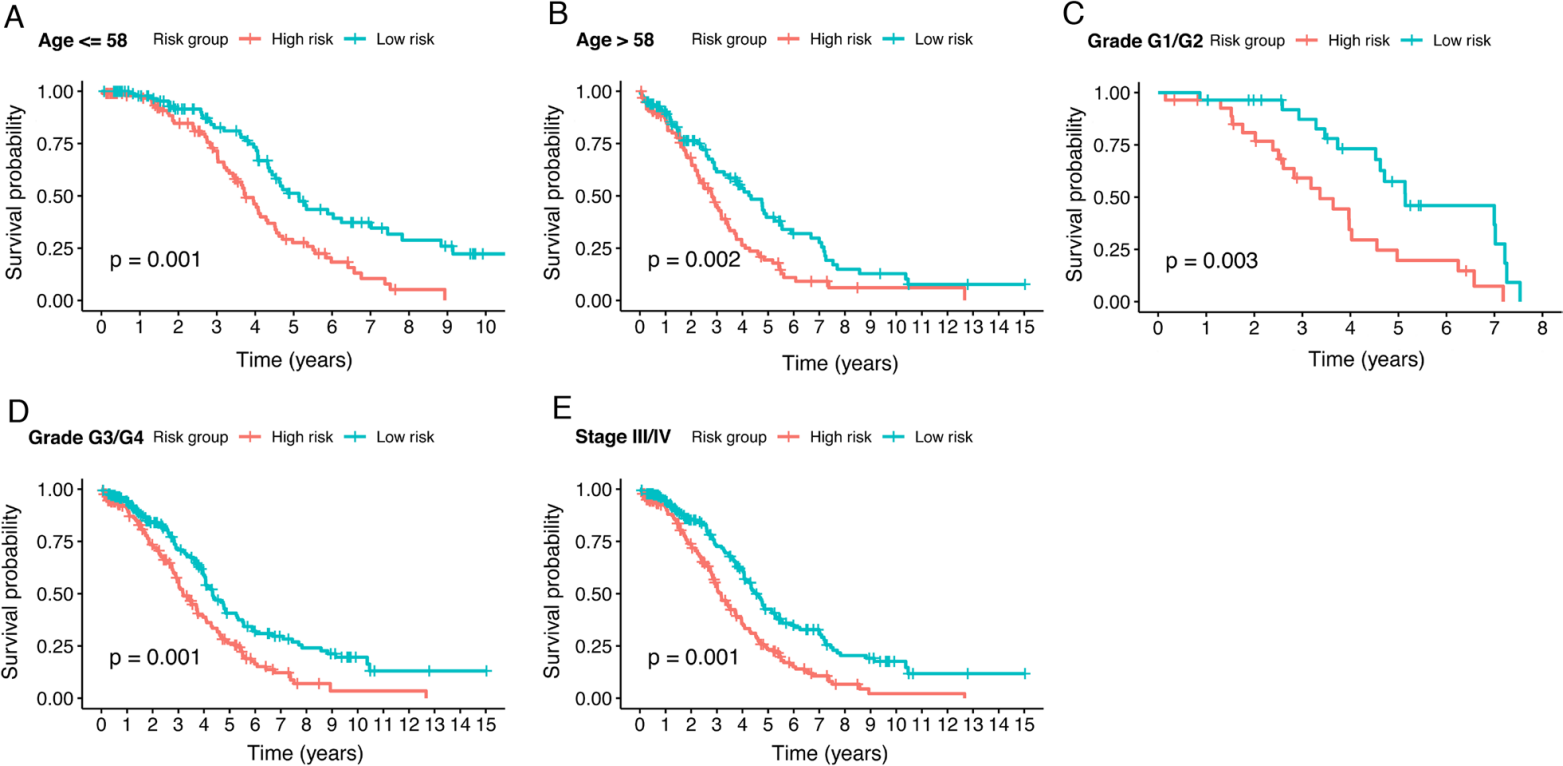
Survival analysis of the risk model by clinical characteristics. Kaplan–Meier plots for OS of several clinical features based on the risk model (log-rank test). **A** Age ≤ 58. **B** Age > 58. **C** Grade G1/G2. **D** Grade G3/G4. **E** Stage III/IV

The whole patient group (394 patients) was further classified according to age, tumor grade, and tumor stage to verify the prognostic value of the risk model. In patients younger than 58 years old, the high-risk group had significantly shorter OS (p = 0.001) (Fig. 4A). Similar results were observed in patients over 58 years old, wherein the high-risk group had worse survival outcomes (*p* = 0.002) (Fig. 4B). In accordance with the results in grade 1/2 patients, an elevated risk score in grade 3/4 patients predicted unfavorable survival prognosis (*p* = 0.003 and *p* = 0.001, respectively) (Fig. 4C, D). By survival analysis in the stage III/IV patients, the results were identical. Patients with elevated risk score had poorer OS (*p* = 0.001) (Fig. 4E). Together, this risk model could be used to predict the survival prognosis of ovarian cancer patients.

### Predictive power of the risk model on survival

**Fig. 5:**
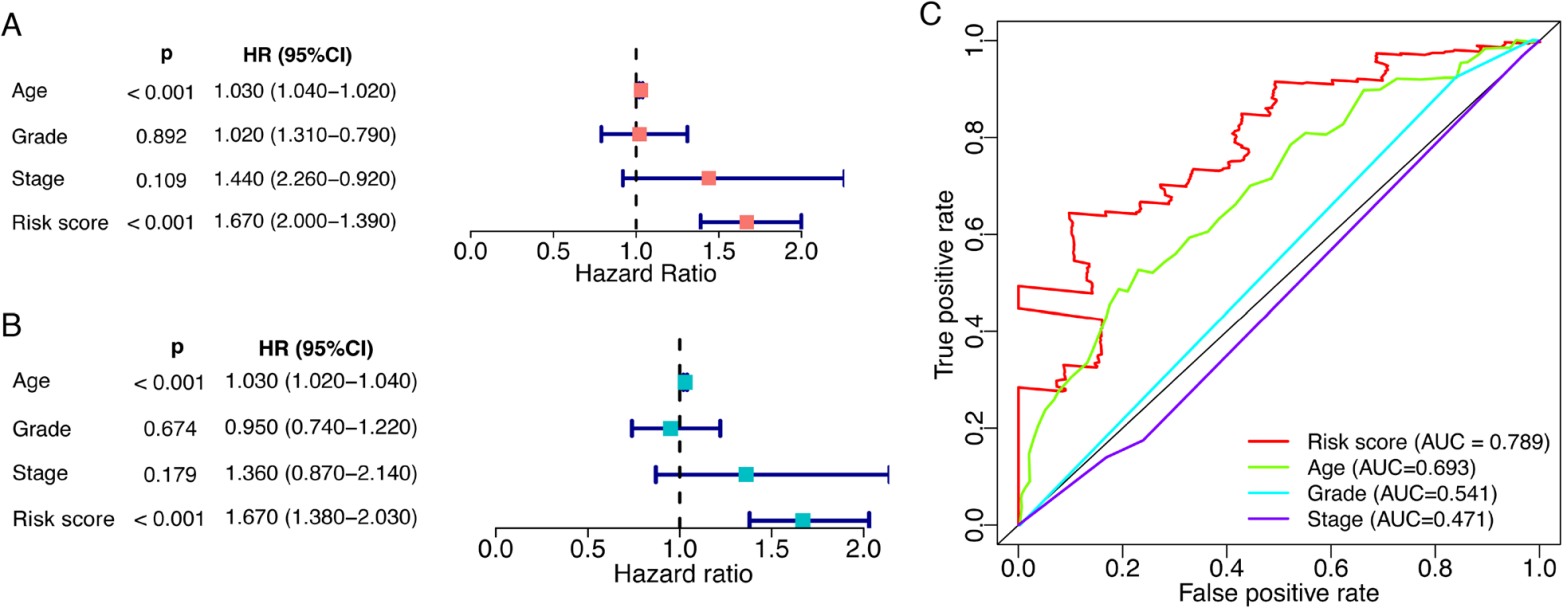
Predictive power comparison of the risk model with other clinical factors. **A** Forest plot of univariate Cox regression analysis for the risk model and several different clinical factors on OS. **B** Multiple Cox regression results on OS. **C** Receiver operating characteristic curves of the risk model and clinical features

To investigate the predictive power of our risk model, univariate and multiple Cox regression analysis of the risk score together with clinical characteristics were performed. In univariate Cox regression analysis, both age and our risk model showed statistical significance (*p* < 0.001), wherein older age and higher risk score were hazardous for patients’ survival (Fig. 5A). In multiple Cox regression analysis, age with a hazard ratio (HR) of 1.030 [95% confidence interval (CI), 1.020–1.040] had significance (*p* < 0.001), while the risk model with a HR of 1.670 (95% CI 1.380–2.030) also reached significance (*p* < 0.001) (Fig. 5B). To evaluate the predictive performance of the risk model and clinical variables, ROC curves were drawn and the area under the curve (AUC) was calculated to quantify their predictive power (Fig. 5C). Surprisingly, the risk model had a much higher AUC value than age (AUC: risk score 0.789; age 0.693). These results suggested that our risk model could predict the survival of ovarian cancer patients efficiently.

### Enrichment analysis based on the proteins comprising the risk model

**Fig. 6:**
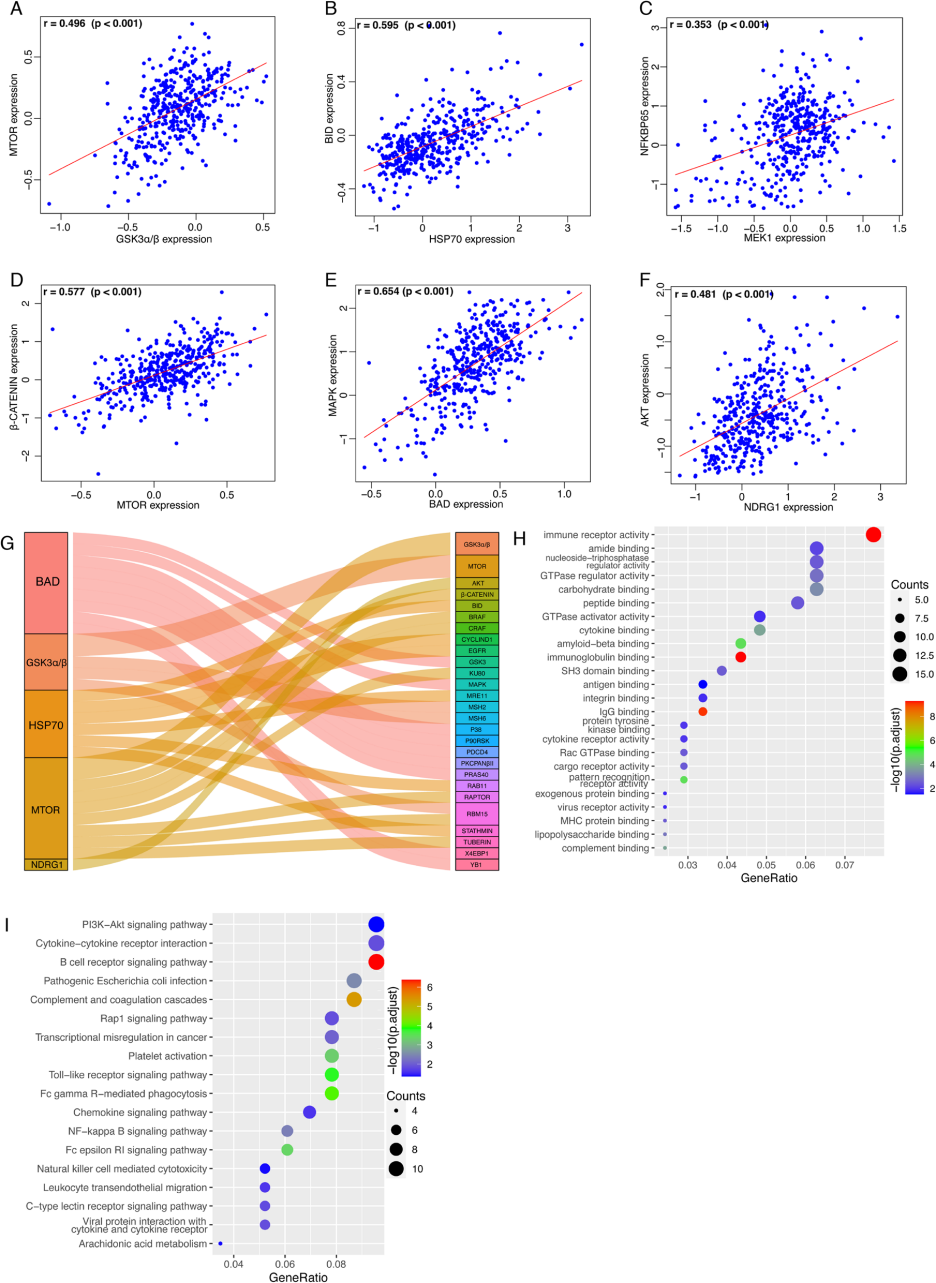
Co-expressed proteins analysis and enrichment analysis based on the risk model. Proteins that are co-expressed with the six proteins comprising the risk model were identified and those having the smallest p value were presented (Pearson’s correlation test). **A** GSK3α/β. **B** HSP70. **C** MEK1. **D** MTOR. **E** BAD. **F** NDRG1. **G** Sankey diagram summarizing co-expressed proteins with the six proteins comprising the risk model. The co-expressed proteins were mapped to their encoding genes, and the co-expressed genes were used for enrichment analysis. **H** GO pathway enrichment analysis of the co-expressed genes. **I** KEGG pathways enriched with the co-expressed genes

To further understand the biological function of the six components of the risk model, we identified proteins whose expression was significantly correlated with their expression. Among all the identified proteins, those having the smallest p value were presented. GSK3α/β had a significant positive correlation with MTOR (r = 0.496, *p* < 0.001) (Fig. 6A). BID was positively correlated with HSP70 (r = 0.595, *P* < 0.001) (Fig. 6B). MEK1 and NFKBP65 had a significant positive correlation (r = 0.353, *p* < 0.001) (Fig. 6C). The expression of β-CATENIN was significantly related to MTOR expression (r = 0.577, *p* < 0.001) (Fig. 6D). BAD and MAPK had a significant co-expression relationship (r = 0.654, *p* < 0.001) (Fig. 6E). As for NDRG1, the top significant protein was AKT (r = 0.481, *p* < 0.001) (Fig. 6F). A sankey plot was drawn to summarize the co-expression signatures (Fig. 6G). Then, the co-expressed proteins were mapped to their encoding genes, and the co-expressed genes were used for enrichment analysis. By GO analysis, GO pathways including immune receptor activity were significantly enriched (Fig. 6H). By KEGG analysis, the co-expressed genes were significantly accumulated in KEEG pathways including PI3K-Akt signaling pathway, B cell receptor signaling pathway, and transcription mis-regulation in cancer (Fig. 6I).

### Functional analysis based on the risk model in ovarian cancer patients

**Fig. 7:**
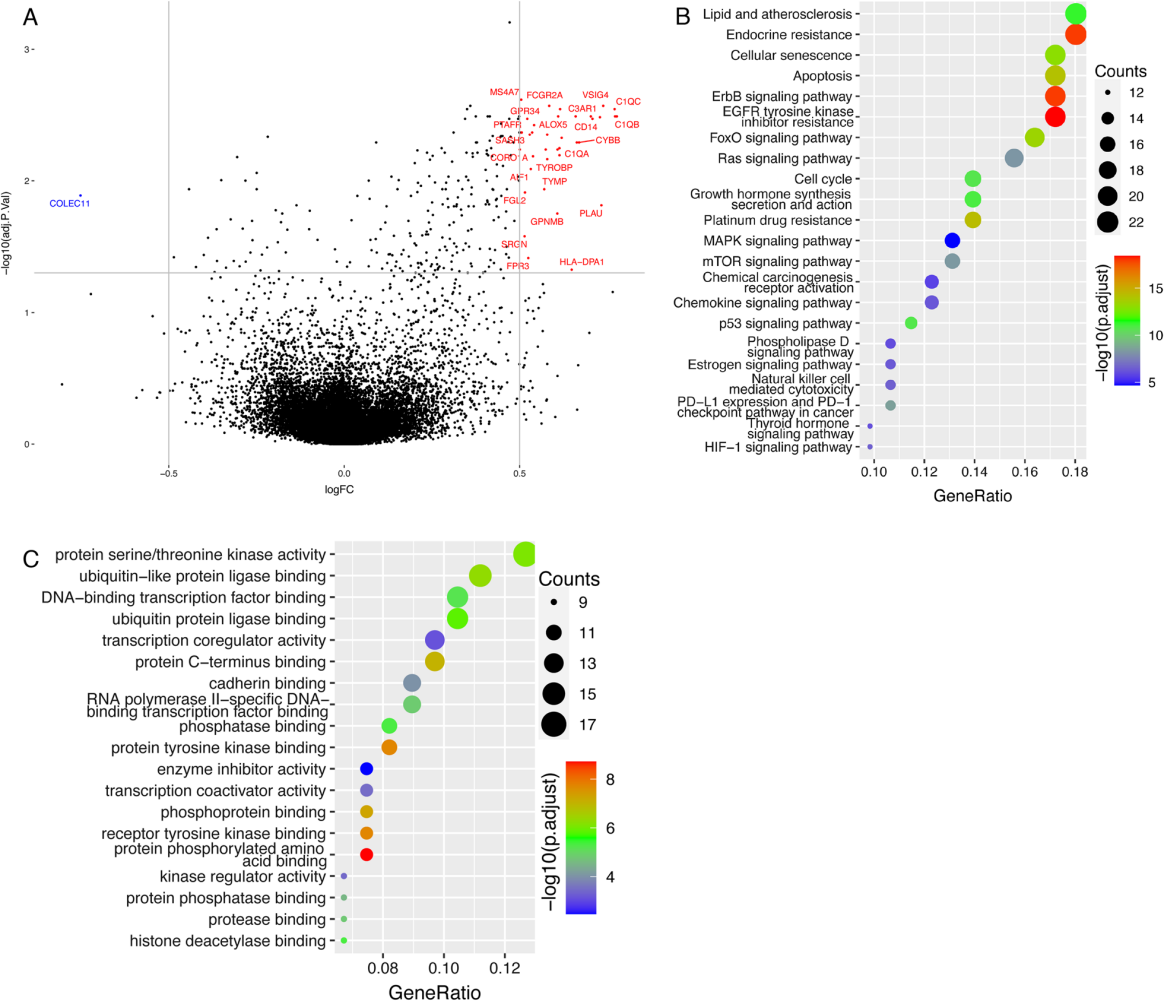
Analysis of differentially expressed genes between the low-risk and high-risk groups. All included ovarian cancer patients were divided into a high-risk group and a low-risk group based on the median risk score. Gene expression was compared between these two groups. **A** Volcano plot showing differentially expressed genes between the low-risk and high-risk groups. A total of 271 differentially expressed genes (adjusted *p* < 0.05) were included in enrichment analysis. **B** KEGG pathways enrichment analysis of the differentially expressed genes. **C** GO pathways enriched with the differentially expressed genes

We further characterized the gene expression profile of ovarian cancer patients based on their risk score to find expression differences. All 394 patients were divided into a high-risk group and a low-risk group based on the median risk score. Gene expression was compared between these two groups and displayed in a volcano plot (Fig. 7A). COLEC11 was significantly downregulated in the high-risk patients, while FCGR2A, CD14, and some other genes were upregulated. A total of 271 differentially expressed genes (adjusted *p* < 0.05) were included in enrichment analysis. These differentially expressed genes were significantly accumulated in KEGG pathways including cellular senescence, apoptosis, cell cycle, platinum drug resistance, and p53 signaling pathway (Fig. 7B). By GO enrichment analysis, GO pathways including protein serine/threonine kinase activity were significantly enriched (Fig. 7C).

## Discussion

Development of prognosis models for ovarian cancer constitutes an important part of risk evaluation and disease management and has a pivotal clinical significance [6]. Here, using the data archived in the TCPA and TCGA databases, we identified a total of 20 proteins associated with OS of ovarian cancer patients using univariate Cox regression analysis. Based on multiple Cox regression analysis, six proteins, namely, GSK3α/β, HSP70, MEK1, MTOR, BAD, and NDRG1, were used for model construction. The risk model could predict OS and DSS of ovarian cancer patients and had higher predictive power than age, tumor grade, and tumor stage. Consistently, all these six proteins were also found to affect OS and DSS of ovarian cancer patients. Functional analyses based on these six proteins and the risk model further supported the prognostic value of our model.

As a promising high-throughput molecular identification method, prognostic biomarker screening based on gene expression profiles can provide patients with accurate prognosis [4, 5]. Our risk model, composed of six proteins (GSK3α/β, HSP70, MEK1, MTOR, BAD, and NDRG1), can predict survival of ovarian cancer patients effectively, thereby helping prognosis monitoring and decision making. GSK3α/β is a serine-threonine kinase and plays a role in glycogen metabolism and neurogenesis. GSK3α/β was found to be involved in tumor growth of ovarian cancer [24]. HSP70 has been correlated with poor prognosis of several cancers, such as cervical cancer, melanoma, gastric cancer, and prostate cancer, and could elicit a strong autoantibody response in ovarian cancer, serving as a tumor-associated antigen [25]. MEK1 acts as a gatekeeper of the MAPK pathway, controlling cell proliferation, differentiation, and therapeutic resistance. MEK1 was reported to be intensely involved in ovarian cancer and has now been explored as a drug target [26]. MTOR is a kind of phosphatidylinositol kinase-related kinase, which can mediate cell response to stress, control cell growth and proliferation, and promote cell survival and cell cycle progression. MTOR has been investigated as a treatment vulnerability in ovarian cancer [27]. BAD, BCL2 associated agonist of cell death, participates in programmed cell death and has proapoptotic ability. BAD was closely related to apoptosis induction and cisplatin responsiveness in ovarian cancer [28]. NDRG1 functions in stress response, hormone response, cell growth, and differentiation, and participates in p53-mediated caspase activation and apoptosis. In ovarian cancer, increased NDRG1 could enhance drug sensitivity through induction of hypoxic stress response [29]. Our results demonstrated that HSP70 was a protective factor for the survival of ovarian cancer patients, high expression of HSP70 indicated favorable OS and DSS. The other five proteins included in the risk model, namely, GSK3α/β, MEK1, MTOR, BAD, and NDRG1, exerted deleterious effects on survival, wherein high expression levels predict poor survival outcomes. The risk model constructed using these six prognostic proteins, which could be detected easily and conveniently by immunohistochemical staining, could predict OS and DSS of ovarian cancer patients efficiently, thereby aiding in patient stratification and disease management.

Prognosis of ovarian cancer is orchestrated by many factors such as age, pathological stage, and histological grade, which remain to be prominent prognostic evaluation tools in clinical application [1, 5]. Tumor stage and grade have been recommended as independent factors for ovarian cancer prognosis [1, 4]. However, in univariate and multiple Cox regression analysis assessing the predictive power of the risk model, tumor stage and tumor grade did not reach the predefined threshold of significance. The incidence of ovarian cancer increases with age [30]. In the present study, both age and the risk model showed predictive power for the survival of ovarian cancer in Cox regression analyses. The risk model displayed an extraordinary performance with an AUC value of 0.789, better than age. Therefore, besides traditional clinicopathological indicators (including age, tumor stage, and tumor grade), our risk model based on protein expression signatures can potentiate accurate prognosis monitoring, thereby facilitating individualized precision medicine, which deserves further exploration and clinical evaluation.

Screening for prognostic biomarkers is of immense benefit to ovarian cancer patients [4]. Nowadays, some extensively used tumor biomarkers in standard clinical practice like CA125 and HE4 lack sensitivity and specificity in disease surveillance [13]. Owing to the prosperous advances in scientific techniques, many genes have been found to harness the potential of prognosis management such as PAWR, RAD51, and AOX1 [7, 8, 31]. Extracellular vesicles-derived miRNAs and proteins like miR-21 and HSP21 can be used in early detection of ovarian cancer [32]. However, a single value is not usually sufficiently accurate and has low predictive performance, since gene expression could have been regulated by various signaling pathways [5]. Applying hub genes that function in signaling transduction to develop multi-gene-based models is essential to facilitate prognosis monitoring and new therapeutic investigation [5]. Some prognostic gene signatures were also explored in ovarian cancer. There were models consisting of autophagy-related genes, ferroptosis-related genes, hypoxia-related genes, immune-related genes, metabolism- and immune-related genes, and RNA binding proteins [4–6, 33–36]. These researches focused on specific aspects of organic physical activities. A seven-gene model was found to predict the prognosis of ovarian cancer, which used the mRNA expression data in the TCGA database and did not include protein expression information [37]. Our risk model comprising of GSK3α/β, HSP70, MEK1, MTOR, BAD, and NDRG1 exploited the data archived in TCPA and TCGA databases and could be utilized as a prognostic indicator.

Co-expression analysis of the six proteins comprising the risk model has assisted in understanding their biological functions, further revealing the implication of the risk model in ovarian cancer. Co-expressed proteins like BID, NFKBP65, β-CATENIN, MAPK, and AKT play important role in ovarian cancer. GO and KEGG pathways enriched with co-expressed genes also exert effects in ovarian cancer, for example, the PI3K-Akt signaling pathway regulates cell proliferation, apoptosis, metastasis, and chemo-resistance of ovarian cancer [38]. Functional analysis based on the risk model also implied its significance in ovarian cancer. The significantly differentially expressed genes could play roles in ovarian cancer. COLEC11 functions in innate immunity and apoptosis [39]. FCGR2A affects the drug response of ovarian cancer [40]. CD14 is associated with ovarian cancer progression [41]. Besides, results of the pathway enrichment analysis profoundly suggested the vital effects of the risk model in ovarian cancer. Cellular senescence, apoptosis, cell cycle, platinum drug resistance, and p53 signaling pathway are all occupied in ovarian carcinogenesis. Similar risk models were also studied in lung cancer, endometrial cancer, and stomach cancer [42–44]. However, our study has some limitations. The TCPA data collection only contains expression data of 223 proteins, which represents only a small portion of the whole protein pool. Besides, the sample size was relatively small and there was no external validation, which could bring in some bias.

## Conclusions

In summary, we analyzed the protein expression profile of ovarian cancer and developed a prognostic model based on univariate and multiple Cox regression analysis. The risk model composed of six proteins (GSK3α/β, HSP70, MEK1, MTOR, BAD, and NDRG1) could predict the survival prognosis of ovarian cancer efficiently and had a prominent predictive performance. The six proteins and the risk model were principally involved in ovarian cancer and had versatile biological functions. Our study adds evidence to prognosis monitoring, provides possibility for future research, facilitates individualized disease management, and warrants further validation and deeper exploration.

## Supplementary Information

**Additional file 1: Fig. S1.** Validation of the risk model in the entire patient group. Ovarian cancer patients were divided into a high-risk group and a low-risk group based on the median risk score. **A** Kaplan–Meier survival curves for OS in all included ovarian cancer patients (log-rank test). **B** Risk score distribution for all ovarian cancer patients in the high-risk and the low-risk groups. **C** Survival status of all included ovarian cancer patients. **D** The expression profiles of the six proteins comprising the risk model in all ovarian cancer patients.

**Additional file 2: Fig. S2**. Prognostic effect of the risk model and the six composing proteins on DSS. **A** Kaplan–Meier plot for DSS of all included ovarian cancer patients grouped by median risk score (log-rank test). Survival analysis of DSS comparing the high and low levels of the six proteins comprising the risk model (log-rank test). **B** GSK3α/β. **C** HSP70. **D** MEK1. **E** MTOR. **F** BAD. **G** NDRG1.

## Data Availability

The datasets supporting the conclusions of this article are available in the TCGA data portal (https://portal.gdc.cancer.gov/) and the TCPA data portal (https://tcpaportal.org/tcpa).

## Abbreviations

AUC: Area under the curve
DSS: Disease-specific survival
GO: Gene Ontology
KEGG: Kyoto Encyclopedia of Genes and Genomes
OS: Overall survival
ROC: Receiver operating characteristic curve
TCGA: The Cancer Genome Atlas
TCPA: The Cancer Proteome Atlas
