# Correction: Protein expression profiling identifies a prognostic model for ovarian cancer

**DOI:** 10.1186/s12905-025-03703-5

**Published:** 2025-04-12

**Authors:** Luyang Xiong, Jiahong Tan, Yuchen Feng, Daoqi Wang, Xudong Liu, Yun Feng, Shusheng Li

**Affiliations:** 1 Department of Critical Care Medicine, Tongji Hospital, Tongji Medical College, Huazhong University of Science and Technology, Wuhan, China; 2 Department of Obstetrics and Gynecology, National Key Clinical Specialty of Gynecology, the First People’S Hospital of Yunnan Province, the Affiliated Hospital of Kunming University of Science and Technology, Kunming, China; 3 Division of Pulmonary and Critical Care Medicine, Department of Internal Medicine, Tongji Hospital, Tongji Medical College, Huazhong University of Science and Technology, Wuhan, China; 4 Department of Urology, The Second Affiliated Hospital of Kunming Medical University, Kunming, China; 5 Department of Pancreatic Surgery, Wuhan Union Hospital, Tongji Medical College, Huazhong University of Science and Technology, Wuhan, China

**BMC Women’s Health 22, 292 (2022)**

**https://doi.org/10.1186/s12905-022–01876-x**

In the funding section of this article [1], the grant number for "Doctoral Research Fund Program of the First People’s Hospital of Yunnan Province” was missing and should have been KHBS-2022-026.

In addition, the grant number for ‘National Key Clinical Specialty of Gynecology・First People’s Hospital of Yunnan Province’ was incorrectly given as ‘2022FKZDZK-18’ and should have been ‘2022FKZDZK-16’. The corrected funding section is given below. The original article has been corrected.

Funding

This work was supported by Yunnan Provincial Clinical Medical Center (2022LCZXKF-SZ08), National Key Clinical Specialty of Gynecology First People’s Hospital of Yunnan Province (2022FKZDZK-16), the Doctoral Research Fund Program of the First People’s Hospital of Yunnan Province (KHBS-2022–026), and Development Center for Medical Science & Technology of National Health Commission (HDSL202003003).
