# Supplementary material for: Protein expression profiling identifies a prognostic model for ovarian cancer
Source: BMC Womens Health. 2022 Jul 15;22:292. doi: 10.1186/s12905-022-01876-x (PMC9284690; doi:10.1186/s12905-022-01876-x)
Supplement: Supplementary file 1 — Additional file 1: Fig. S1. Validation of the risk model in the entire patient group. Ovarian cancer patients were divided into a high-risk group and a low-risk group based on the median risk score. A Kaplan–Meier survival curves for OS in all included ovarian cancer patients (log-rank test). B Risk score distribution for all ovarian cancer patients in the high-risk and the low-risk groups. C Survival status of all included ovarian cancer patients. D The expression profiles of the six proteins comprising the risk model in all ovarian cancer patients. [file 12905_2022_1876_MOESM1_ESM.docx]

**
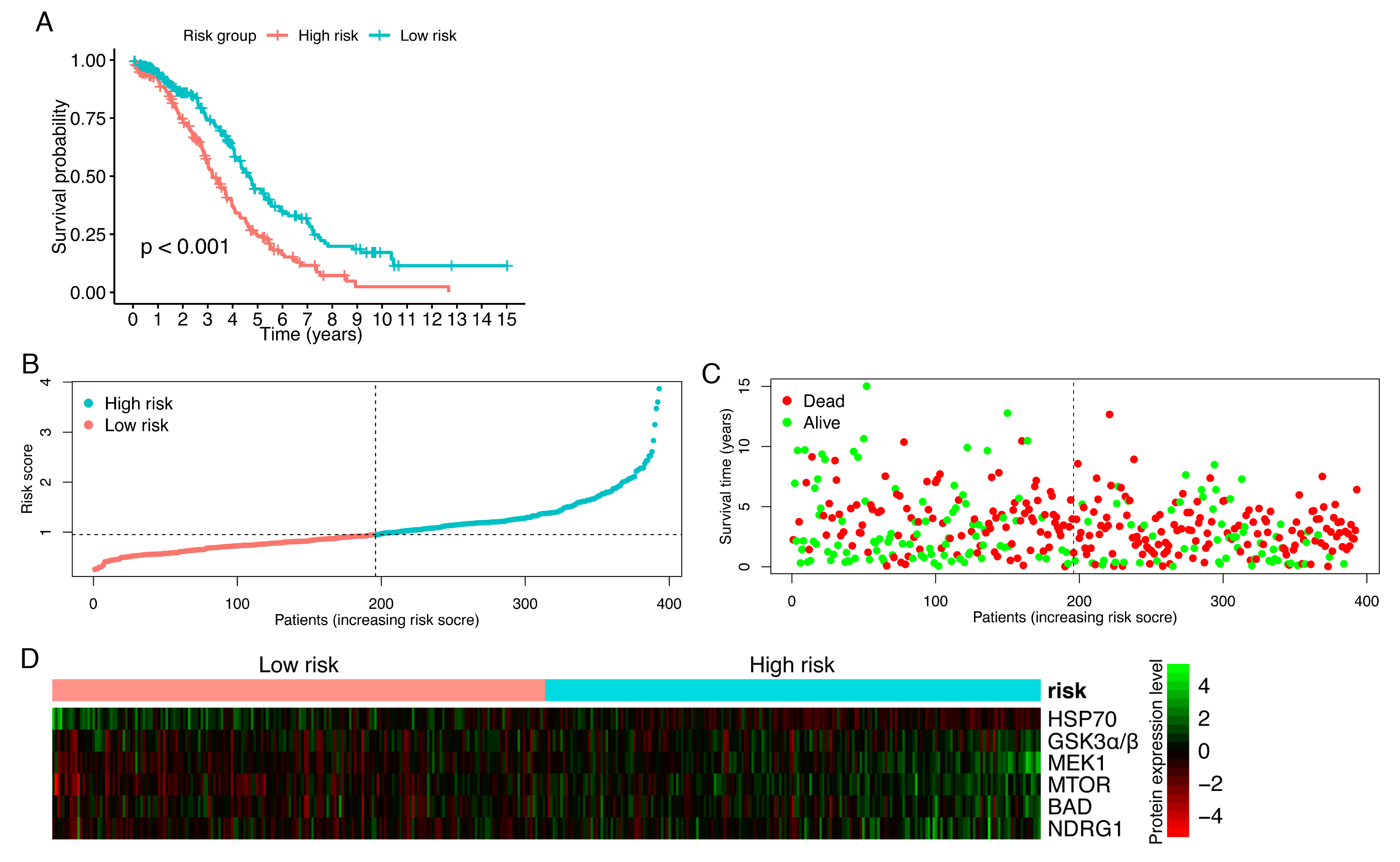
**

**Additional file 1:** Supplementary figure s1. Validation of the risk model in the entire patient group. Ovarian cancer patients were divided into a high-risk group and a low-risk group based on the median risk score. **A.** Kaplan-Meier survival curves for OS in all included ovarian cancer patients (log-rank test). **B.** Risk score distribution for all ovarian cancer patients in the high-risk and the low-risk groups. **C.** Survival status of all included ovarian cancer patients. **D.** The expression profiles of the six proteins comprising the risk model in all ovarian cancer patients. (TIFF).
