# Supplementary material for: Protein expression profiling identifies a prognostic model for ovarian cancer
Source: BMC Womens Health. 2022 Jul 15;22:292. doi: 10.1186/s12905-022-01876-x (PMC9284690; doi:10.1186/s12905-022-01876-x)
Supplement: Supplementary file 2 — Additional file 2: Fig. S2. Prognostic effect of the risk model and the six composing proteins on DSS. A Kaplan–Meier plot for DSS of all included ovarian cancer patients grouped by median risk score (log-rank test). Survival analysis of DSS comparing the high and low levels of the six proteins comprising the risk model (log-rank test). B GSK3α/β. C HSP70. D MEK1. E MTOR. F BAD. G NDRG1. [file 12905_2022_1876_MOESM2_ESM.docx]

**
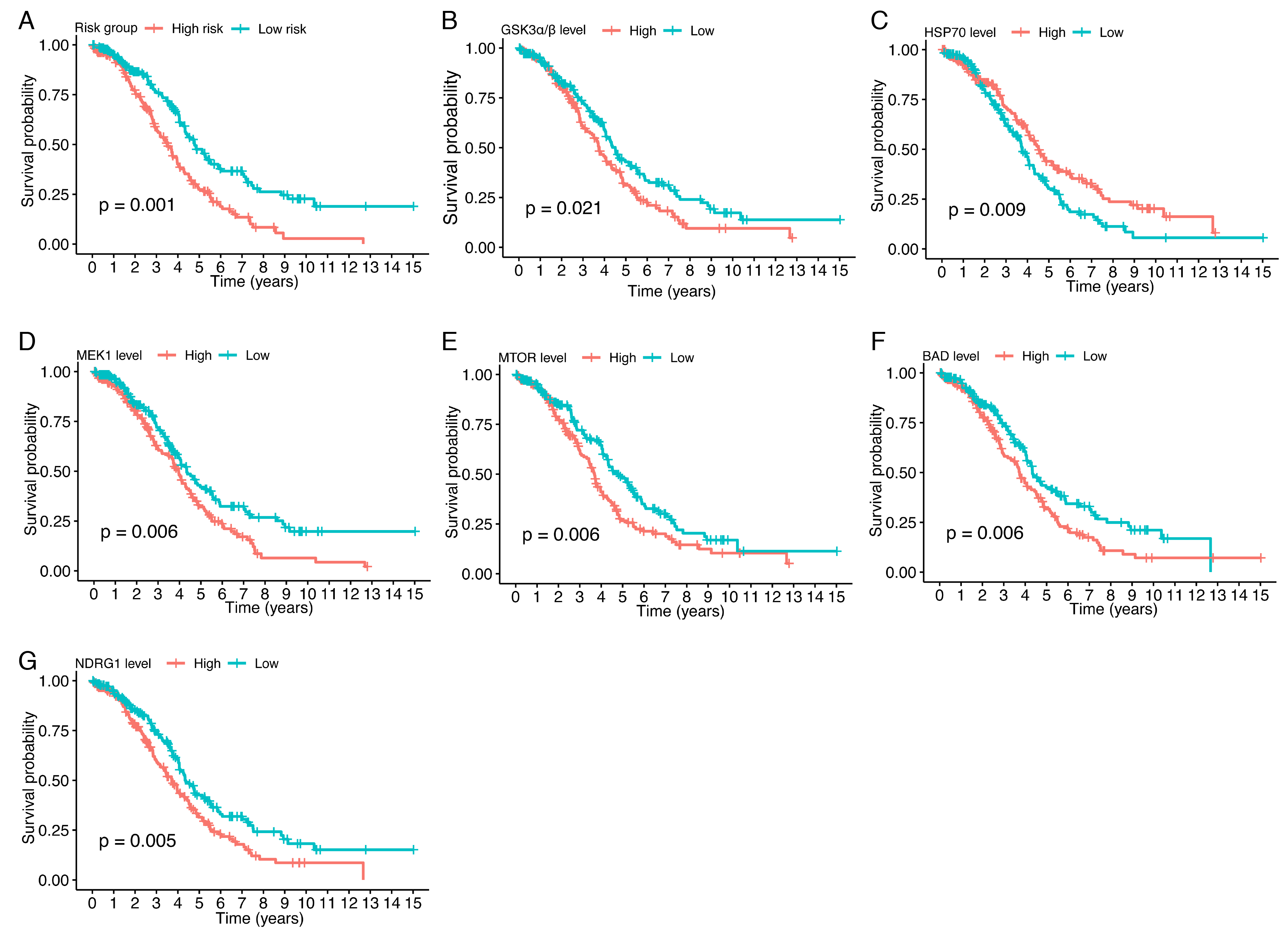
**

**Additional file 2:** Supplementary figure s2. Prognostic effect of the risk model and the six composing proteins on DSS. **A.** Kaplan-Meier plot for DSS of all included ovarian cancer patients grouped by median risk score (log-rank test). Survival analysis of DSS comparing the high and low levels of the six proteins comprising the risk model (log-rank test). **B.** GSK3α/β. **C.** HSP70. **D.** MEK1. **E.** MTOR. **F.** BAD. **G.** NDRG1. (TIFF)
